# An Index for Lifting Social Distancing During the COVID-19 Pandemic: Algorithm Recommendation for Lifting Social Distancing

**DOI:** 10.2196/22469

**Published:** 2020-09-17

**Authors:** Sam Li-Sheng Chen, Amy Ming-Fang Yen, Chao-Chih Lai, Chen-Yang Hsu, Chang-Chuan Chan, Tony Hsiu-Hsi Chen

**Affiliations:** 1 College of Oral Medicine Taipei Medical University Taipei Taiwan; 2 Institute of Epidemiology and Preventive Medicine College of Public Health National Taiwan University Taipei Taiwan; 3 Institute of Environmental and Occupational Health Science College of Public Health National Taiwan University Taipei Taiwan

**Keywords:** COVID-19, pandemic, social distancing, index, algorithm, data analysis, decision making, global health, public health

## Abstract

**Background:**

Implementing and lifting social distancing (LSD) is an urgent global issue during the COVID-19 pandemic, particularly when the travel ban is lifted to revive international businesses and economies. However, when and whether LSD can be considered is subject to the spread of SARS-CoV-2, the recovery rate, and the case-fatality rate. It is imperative to provide real-time assessment of three factors to guide LSD.

**Objective:**

A simple LSD index was developed for health decision makers to do real-time assessment of COVID-19 at the global, country, region, and community level.

**Methods:**

Data on the retrospective cohort of 186 countries with three factors were retrieved from a publicly available repository from January to early July. A simple index for guiding LSD was measured by the cumulative number of COVID-19 cases and recoveries, and the case-fatality rate was envisaged. If the LSD index was less than 1, LSD can be considered. The dynamic changes of the COVID-19 pandemic were evaluated to assess whether and when health decision makers allowed for LSD and when to reimplement social distancing after resurgences of the epidemic.

**Results:**

After large-scale outbreaks in a few countries before mid-March (prepandemic phase), the global weekly LSD index peaked at 4.27 in March and lasted until mid-June (pandemic phase), during which most countries were affected and needed to take various social distancing measures. Since, the value of LSD has gradually declined to 0.99 on July 5 (postpandemic phase), at which 64.7% (120/186) of countries and regions had an LSD<1 with the decile between 0 and 1 to refine risk stratification by countries. The LSD index decreased to 1 in about 115 days. In addition, we present the results of dynamic changes of the LSD index for the world and for each country and region with different time windows from January to July 5. The results of the LSD index on the resurgence of the COVID-19 epidemic in certain regions and validation by other emerging infectious diseases are presented.

**Conclusions:**

This simple LSD index provides a quantitative assessment of whether and when to ease or implement social distancing to provide advice for health decision makers and travelers.

## Introduction

Although border controls and social distancing have been executed since the beginning of the COVID-19 pandemic [1-4], we must consider how to lift social distancing in the postpandemic period with real-time assessment, possibly weekly [5], as reviving economic business and normal social activities are needed.

Although there are six criteria for countries to consider when de-escalating by reversing restrictions or lockdowns [6], it is still unclear whether and when to implement the reopening policy. Doing so is highly dependent on three determinants. The first is to consider the transmission rate of COVID-19 that is often captured by the basic reproductive number (R_0_; the expected number of secondary cases produced by an index case in a susceptible population) using the susceptible- infected- recovered or susceptible- exposed- infected- recovered models to evaluate the effectiveness of social distancing [2-4,7-11]. The second is pertaining to the optimal management of patients with COVID-19 that determines the rate of recovery from hospitalization or self-isolation. The third is strongly related to the second determinant and critical care capacity, and concerns preventing mild or moderate patients from deteriorating into severe patients, potentially causing further death from COVID-19. A simple lifting social distancing (LSD) index is required to quantify the impacts of these three factors on LSD. The implications for developing an LSD index are two-fold. From a global perspective, providing this LSD index can aid policy makers worldwide in evaluating and deciding when to reopen the border if the spread of SARS-CoV-2 can be contained even with small cluster infections. From an individual viewpoint, if information on this index can be evaluated periodically and incorporated into traveling apps or websites, it would be helpful for the traveler to be aware of information on these three determinants in each country or region to decide whether it is safe for them to travel to their destinations and, if they must travel in the coming weeks, how they can be prepared to protect themselves from being infected with COVID-19 in high LSD areas.

The aim of this study was to develop a simple index for guiding LSD with assessment on the global, country, region, and community level from January until early July.

## Methods

### Data

The data for the following analysis were derived from the web-based real-time GitHub repository created by the Center for Systems Science and Engineering (CSSE) at Johns Hopkins University [12,13]. CSSE operates daily updates of publicly available data, including confirmed cases, recovered cases, and deaths from multiple sources. A total of 186 countries have reported confirmed COVID-19 cases (including presumptive positive cases and probable cases) at the country and region level, which are aligned with the World Health Organization (WHO) situation reports [14]. A total of 186 affected countries and regions were available from January 21 to July 5, 2020.

These three factors (cases, recoveries, and deaths), available from open data, were used as proxy variables for the corresponding three metrics, including transmissibility that is often captured by the R_0_, number of beds for hospitalization, and number of beds for the intensive care unit. However, the two latter metrics may not be available from open data in the three periods (prepandemic, pandemic, and postpandemic), described in the next section, from a global perspective.

### Social Distancing and Three Factors Related to COVID-19

At the beginning of the outbreak, each country focused on the evaluation of the spread of COVID-19, which often refers to the evolution of the R_0_. With time, those accumulated COVID-19 cases have invoked demand for hospitalization and critical care provided for moderate and severe patients, respectively. To reduce the burden of medical resources and disease burden of death, various containment measures would be adopted to stamp out large-scale community-acquired outbreaks. Although different terminologies have been used worldwide, the broad term “social distancing” is used here to represent containment measures and is defined as border control across countries, lockdown across cities, physical distancing between individuals, and use of face masks. Nonetheless, if social distancing lasts for a long time, economic and social activities will be restricted. In the postpandemic period, the majority of countries and regions may still have small outbreaks after large-scale outbreaks. To balance social distancing and economic revival, each country or region has to consider whether the medical capacity can afford newly increasing confirmed COVID-19 cases arising from small outbreaks due to LSD. Assessing three factors (cases, recoveries, and deaths) simultaneously that are reciprocal and correlated with each other provides an insight into the balance between LSD and disease burden.

### Statistical Analysis

#### Index for LSD

We developed the simple index for LSD, which originated from the susceptible-exposed-infected-recovered-death (SEIRD) compartment model for infectious disease [15]. The semantics of SEIRD is denoted by five symbols, including susceptible (S), exposed (E), infected (I), recovered (R), and death (D), as illustrated in the figure in Multimedia Appendix 1. It has been already applied to modeling the dynamic transmission of SARS-CoV-2 [16]. In a mathematical way, let the number of the compartments S, E, I, R, and D at time t be denoted by s(t), e(t), i(t), r(t), and m(t), respectively. Given a time point, participants of a country or region belong to one of the five states. The instantaneous change for the compartments thus constitutes a system of differential equations:

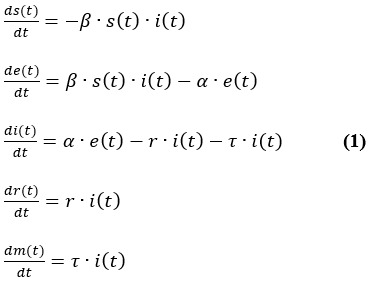



where β represents the transmission coefficient, and α, γ, and τ denote the progression rates to infectious status, recovery rate, and case-fatality rate, respectively.

Based on the system of differential equations in equation 1, the cumulative frequencies of cases, recoveries, and deaths with the consideration of the COVID-19 evolution among a population, given period *t*, can be derived as follows:

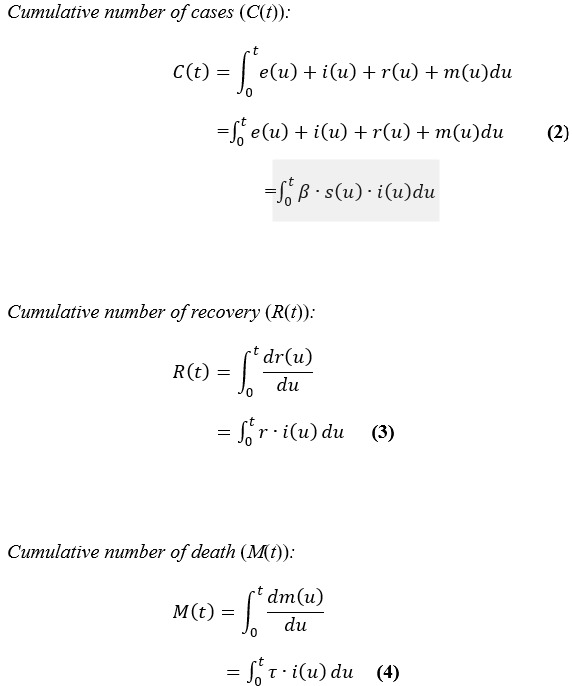



Note that the transmission of COVID-9 captured by the transmission coefficient, β, is altered by social distancing measures that would reduce the frequency of contact and the probability of transmission [17,18], the recovery rate and case-fatality rate that are determined by the optimal management of medical care, the capacity of hospitalization and intensive care, and the quality of care [19]. Without a proper triage and diversion of patients with COVID-19, a compromised recovery rate and an increasing case fatality will be expected [20].

Rooted from the mathematical modeling in dynamics of infectious disease from contact and transmission until recovery or death, we developed an index for LSD, taking into account three elements encrypted in the SEIRD model, namely, the force of disease transmission (*β*), the optimal provision of health care service reflected by the recovery rate (γ), and the critical care capacity captured by the case-fatality rate (τ). Specifically, this can be derived by:

P (Recovery | Infected COVID-19 cases) **(5)**

which can be decomposed into:

P(Recovery| Infected COVID-19 cases and still survive) × **(6)**

P(Survive | Infected COVID-19 cases)

where the first part is the recovery rate and the second part corresponds to the complement of the case-fatality rate. By using the cumulative frequencies specified by equations 2-4, equation 6 can be written as:

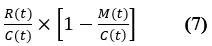



which is equivalent to:

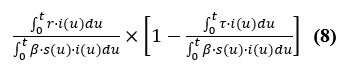



An index for LSD is extended by taking the inverse of equation 8 to have:

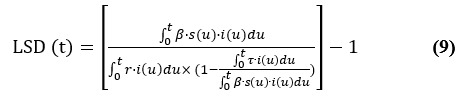



The reason for subtracting 1 is that, in an ideal scenario, the aforementioned ratio would reach 1 when all confirmed cases have been recovered without death (case-fatality rate 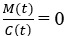), and therefore, LSD would approach zero, suggesting the region has a full recovery after the outbreak of COVID-19 and may return to the normal status.

#### Derivation of LSD From Empirical Data

To fit the formula of LSD(t) in equation 9 with the empirical data, the numerator would be estimated by the cumulative number of cases up to time *t* in empirical data, and the denominator would be derived by the cumulative number of recoveries and the case-fatality rate based on the corresponding date. An index for LSD for time *t* following equation 9 can be explicitly described as follows:

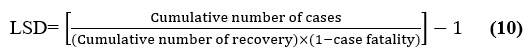



LSD is, thus, the ratio of cumulative confirmed cases to cumulative recovered patients without dying from COVID-19 that is captured by (1 – case fatality) – 1 during a fixed time period.

However, it is impracticable to lift social distancing until the value of LSD reaches 0. One has to consider the balance between the spread of COVID-19, the rate of recovery, and the capacity of critical care. The first element is to capture the information on the rate of the COVID-19 spread after the implementation of social distancing that is often modeled by the R_0_. The second element is dependent on whether health care systems have the capacity to offer a number of beds for hospitalization. The third one is determined by the capacity of critical care that can stop the progression from acute respiratory distress syndrome to death.

It should be noted that three factors are affected by each other and may also allow for other related factors beyond the previously mentioned determinants. For example, if there is an increase in COVID-19 cases due to LSD and if hospital beds have been occupied for other reasons (trauma, elective surgeries, etc), the rate of recovery would be slow, and the case-fatality rate may be higher. This can be captured by the proposed index. Here, we also made the recommendation for LSD using this index. If the value of the LSD index is greater than 1, it is still necessary to maintain social distancing because the rate of the COVID-19 spread still outweighs the affordable capacity of hospitalization and critical care. If it is less than 1, LSD can be suggested. The degree of an LSD less than 1 was assessed by the inverse of the decile of the LSD index from the lowest decile (0.1) to the highest decile (1). The multinomial distribution using the reported number of confirmed cases, recoveries, and deaths in conjunction with the Bayesian Markov chain Monte Carlo method was used for the derivation of 95% credible interval (CI) for the LSD index.

### External Applications of LSD Index to Emerging Infectious Disease

Other emerging infectious disease data including severe acute respiratory syndrome (SARS), Middle East respiratory syndrome–related coronavirus (MERS-CoV), and Ebola were used for the validation of the developed LSD index. The daily reported cases, recoveries, and deaths for the SARS outbreak in 2003 were collected from the Taiwan Centers for Disease Control. Data for the MERS-CoV outbreak in South Korea in 2015 were retrieved from published literature [21]. Data for the Ebola outbreak in the Democratic Republic of Congo in 2018 were derived from the WHO report [22].

## Results

### The Daily LSD Index for Countries and Regions Worldwide

As of July 5, 2020, 11,388,537 confirmed cases, 6,445,646 recoveries, and 533,638 deaths from COVID-19 were reported worldwide, which gave a case-fatality rate of 4.7% and a recovery rate of 57%.

**Figure 1:**
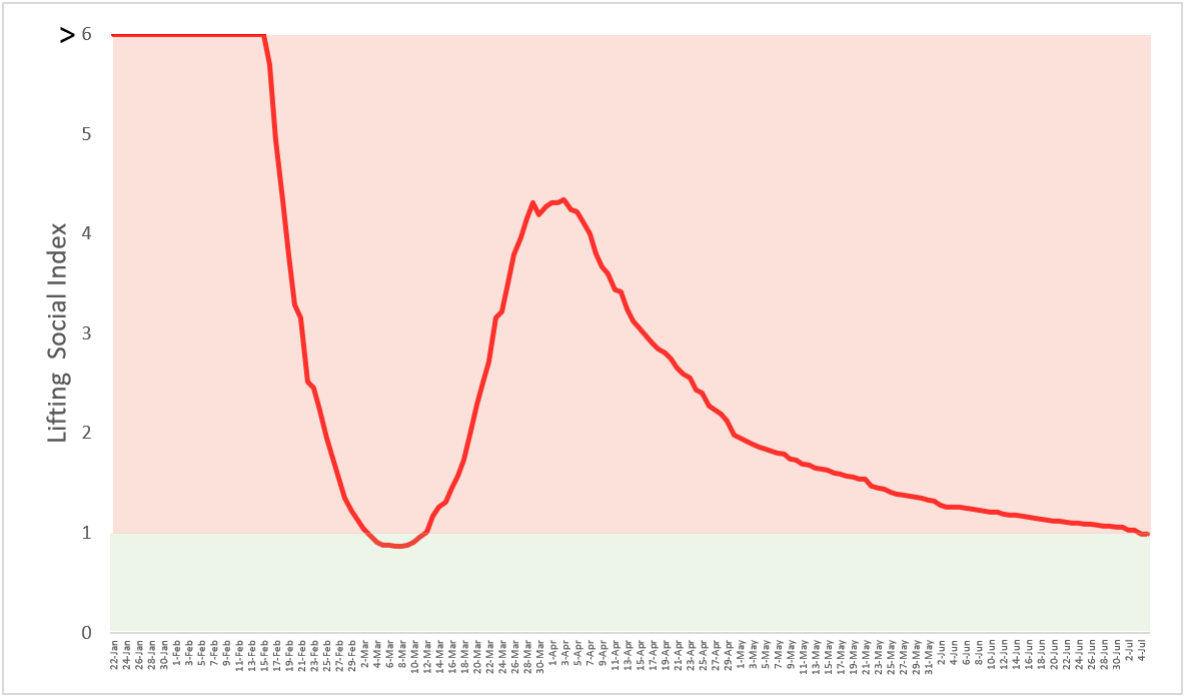
Temporal trend of global lifting social distancing (LSD) index up to July 5, 2020. Overall: The LSD index ranged between 40.1 and 0.96 in the prepandemic period from January to mid-March. In the pandemic period from mid-March to June, the peak of the LSD index reached 4.27 on March 29. As of July 5, the LSD index declined to less than 1.

Figure 1 shows the daily temporal trend of the global LSD index. The global daily LSD index began with 40.84-11.63 between the last week of January and the second week of February, mainly from Mainland China. In the third week of February, it declined but was still large, up to 5.69 (95% CI 5.65-5.73), mainly due to the spread from China to other hot spots including South Korea, Iran, and Italy. The period from January to mid-March, just before the WHO categorized the COVID-19 pandemic as being in the “pre-pandemic phase.” After that, the value of LSD peaked again on March 29 (4.27, 95% CI 4.26-4.28) due to a large-scale spread from continental regions including Australia, the United States and Canada (North America), the European Union except Italy, South Africa, and South Asia. The period from mid-March to June was called the “pandemic phase.” During the pandemic phase, every country had adopted various containment measures, which rendered the value of LSD to gradually decline and fall to 1.11 on June 21 and further decrease to 0.99 by July 5. The phase after June is called the “post-pandemic phase.” The LSD index took 115 days to go down to 1 at the global level.

**Figure 2:**
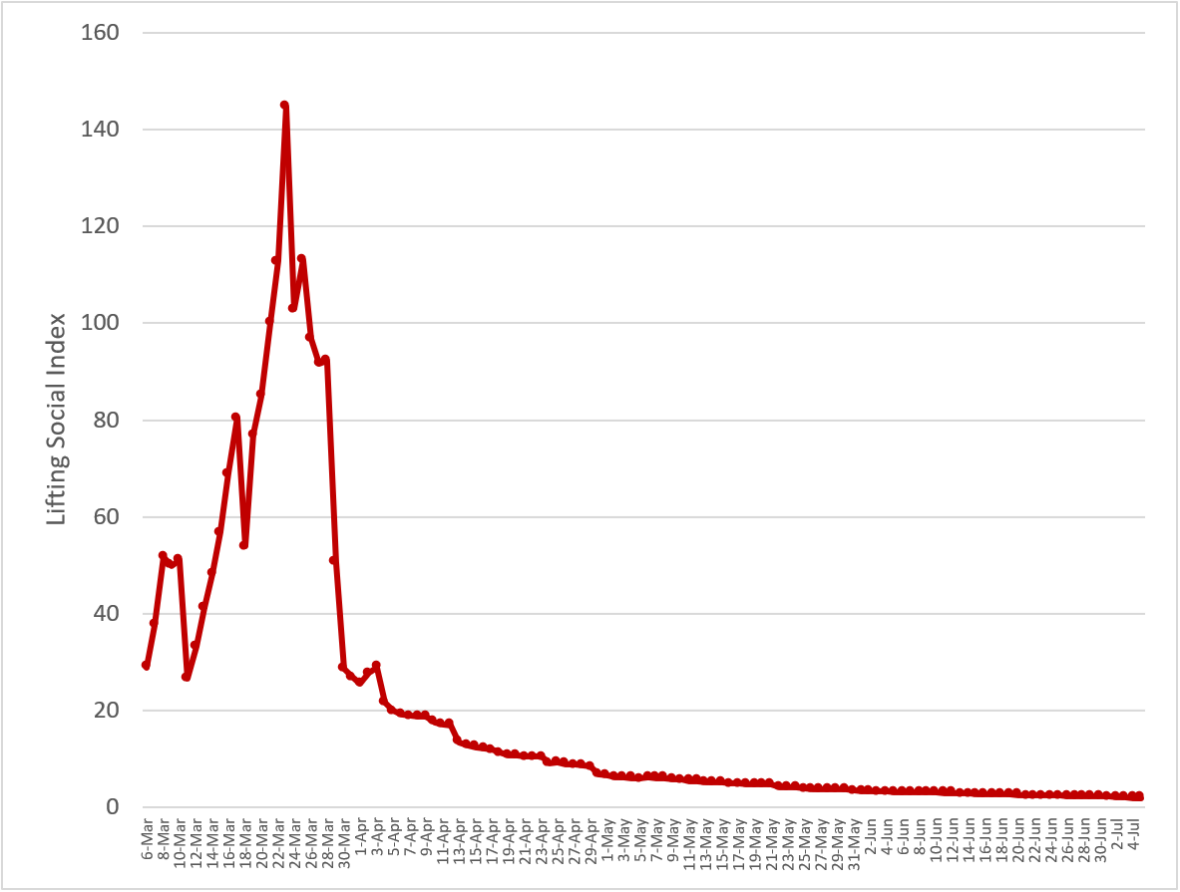
Temporal trend of global lifting social distancing (LSD) index up to July 5, 2020. A total of 66 countries with LSD≥1 on June 28 in the pandemic phase period. The social distancing was relaxed over 4 months.

**Figure 3:**
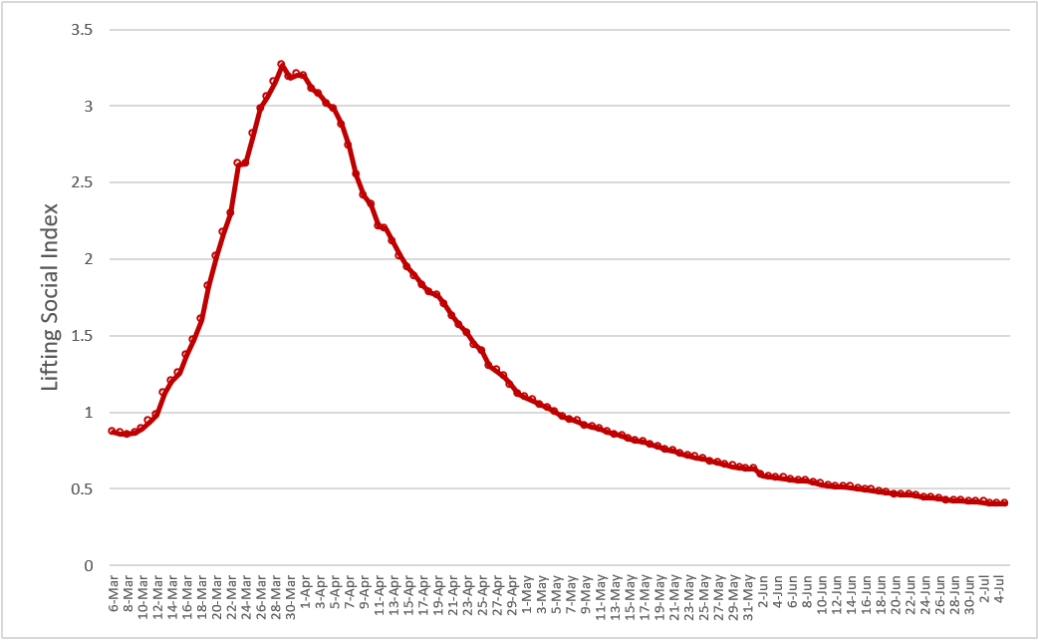
Temporal trend of global lifting social distancing (LSD) index up to July 5, 2020. A total of 120 countries with LSD<1 on June 28 in the pandemic phase. Between March 13 and May 6, the LSD index was greater than 1. The time required for lifting social distancing was 55 days on average.

It should be taken with great caution that the value of LSD was below 1 on July 5, 2020, as the overall curve consisted of most countries with an LSD value smaller than 1 (65%), and the remaining countries (35%) were larger than 1. It is, therefore, necessary to stratify the overall curve into two types according to the LSD value greater than or less than 1 in the previous week of July 5. For countries with LSD values less than 1, it took 55 days, on average, to enable LSD. At the global level, Figure 2 shows that the spread of COVID-19 still exceeded the capacity of hospitalization and critical care for COVID-19 cases, whereas Figure 3 shows the opposite. These heterogeneous findings across countries and regions also suggests that each country and region may experience three phases (pre-epidemic or cluster infection, epidemic, and postepidemic phases) commensurate with the previously mentioned three corresponding phases of the COVID-19 pandemic in different time periods.

**Figure 4:**
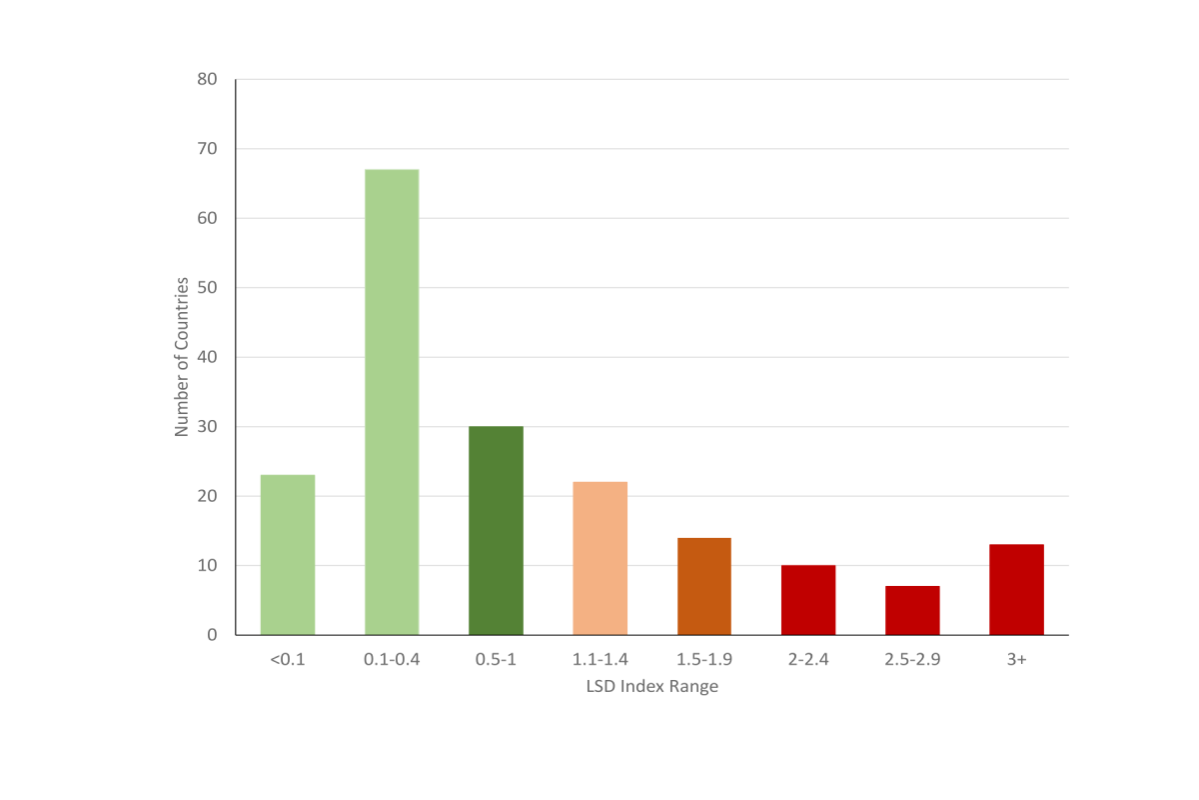
The number of countries and regions by ranges of the LSD index. As of July 5, 2020, the LSD index in 120 countries and regions was less than 1. The LSD index was greater than 1 in 66 countries and regions. LSD: lifting social distancing.

According to the decile of the LSD index for each of the two categories (≥1 and <1), Figure 4 shows the frequencies on the index of LSD until July 5, 2020, for the countries and regions worldwide, aggregated by three groups for the LSD<1, including <0.1 (n=23), 0.1-0.4 (n=67), and 0.5-1 (n=30), and by five groups for the LSD ≥1, including, 1.1-1.4 (n=22), 1.5-1.9 (n=14), 2-2.4 (n=10), 2.5-2.9 (n=7), and ≥3 (n=13). Of the 186 countries and region, 64.5% (n=120) of the LSD indices were less than 1.

### Dynamic Change of Weekly LSD Index Worldwide

Multimedia Appendix 2 shows the dynamic change of the global LSD index (in weeks) for 186 countries and regions from January 26 (fifth week) to July 5, 2020 (28th week), on the map worldwide. Countries that are more red are less likely to lift social distancing, whereas those that are more green are more likely to lift social distancing. Different countries had different times to transit from high (greater than 1) to low (less than 1) LSD values, representing different times required for LSD as indicated in three phases of the COVID-19 epidemic in each country or region. For example, South Korea’s pre-epidemic phase with cluster infections due to religious gatherings was between early February and mid-February. The epidemic phase with large-scale community-acquired outbreaks was between mid-February and March. The postepidemic phase was after March. Various regions had experienced these three phases with different time windows as detailed in Multimedia Appendix 2. The details of the global dynamic change consisting of each country and region have been delineated as follows.

The LSD value in Western European regions had changed from greater than 1 (red) on March 31, 2020, to less than 1 (green) on July 5. However, the evolution and three factors’ contribution were heterogeneous before the decline of the LSD value to being less than 1 between the first week and the fifth week in May. As of the end of May, Germany’s (a lower LSD index of 0.163, 95% CI 0.160-0.166) moderate case-fatality rate (4.7%) was compensated by the high recovery rate (90%), possibly through early detection followed by the high capacity for hospitalization. Countries’ high LSD indices, such as Hungary (LSD: 1.088, 95% CI 1.063-1.114), were attributed to high case-fatality rates (13.6%), mainly resulting from an insufficient capacity for critical care. The high LSD index was, to a greater extent, due to the case-fatality rate (around 2.4 times the average worldwide) and, to a lesser extent, due to a modest recovery rate (55.4%, around 1.3 times the average worldwide). However, the overall value of LSD has declined to 1 since mid-June. Similar findings have been noted in other regions of Europe with different weekly times to see the drop of LSD, including around the fourth week for Northern and Southern Europe, and the fifth week for Eastern Europe. However, the LSD index for a few countries in Southeast Europe was still greater than 1 until mid-June.

Even when there were large-scale outbreaks from mid-March to mid-April that led to the value of LSD being greater than 1 in two Oceania countries including New Zealand and Australia, their values of LSD decreased to less than 1 after both had controlled the outbreak due to multiple containment measures including restricted border control, quarantine, and planned patient care. In addition, the better recovery rate and lower case fatality were also achieved by the optimal management of patients with COVID-19 and the sufficient capacity for critical care.

In the early phase of the COVID-19 pandemic, the spread of SARS-CoV-2 had started from Northeast Asia including South Korea and Japan, and then had subsequent outbreaks in most of the countries in Southeast Asia and South Asia, with the value of LSD greater than 1 in Southeast Asia since mid-March. Some countries including Vietnam, Laos, Cambodia, Thailand, and Myanmar had an LSD value less than 1 after April 19. However, the values of LSD for a few countries such as Indonesia, Philippine, Nepal, India, and Bangladesh were still greater than 1 until July 5.

In Africa, the spread of COVID-19 started in mid-March. After 3 months, the value of LSD was still greater than 1 in most of the countries in Africa until July 5. Similar findings were observed in South Africa, probably resulting from insufficient health care personnel and capacity for critical care.

The LSD index in the United States has not yet reached less than 1 because of 5 states where the impact of high transmission from SARS-CoV-2 and a low recovery rate resulted in higher LSD values in the United States, which has been demonstrated in several states with high long-standing LSD values including New York state, even though the case-fatality rate (6.4%) was comparable to the average worldwide.

Time for LSD in selected countries with an LSD index less than 1 are summarized in Table 1. New Zealand took 22 days as the shortest time to reach an LSD index less than 1. The time for the LSD index to be less than 1 was estimated as 46-51 days for South Korea, Germany, and Australia. Japan and Hungary took longer (around 90 days). Social distancing was lifted while the LSD index was less than 1 in most countries except Finland (1.03), Hungary (1.21), Italy (1.07), Russia (1.03), and Canada (1.03). The Philippines and the United States were too early to lift social distancing. Both of the LSD indices in these two countries were greater than 4.

**Table 1:** Time required and LSD index for LSD in selected countries.

Country	Outbreak date	Date when LSD^a^<1	Time required for LSD (days)	On the date of LSD<1				LSD	
				COVID-19 cases	Recovered cases	Death cases	LSD index	Date	LSD index
New Zealand	March 24	April 15	22	1366	628	9	0.92	April 28	0.22
Vietnam	March 11	April 8	28	251	126	0	0.99	April 24	0.23
Iceland	March 10	April 12	34	1701	889	8	0.92	May 5	0.05
Austria	March 9	April 13	36	131	2	0	64.5	April 28	0.27
Netherlands	March 19	April 27	40	38,440	22,176	4534	0.97	June 2	0.40
Turkey	March 25	May 4	40	127,659	68,166	3461	0.92	June 1	0.31
China	January 22	March 1	41	79,932	42,162	2872	0.97	April 4	0.12
Switzerland	March 3	April 13	41	25,688	13,700	1138	0.96	June 22	0.15
Denmark	March 6	April 18	44	7437	4031	346	0.94	June 8	0.16
Ireland	March 17	April 29	44	20,253	13,386	1190	0.61	May 18	0.33
Australia	March 1	April 17	46	6522	3808	66	0.73	April 28	0.21
Germany	February 27	April 14	47	131,359	68,200	3294	0.98	June 2	0.16
South Korea	February 7	March 29	51	9583	5033	152	0.95	May 6	0.18
Malaysia	February 27	April 15	53	5072	2647	83	0.95	June 9	0.21
Belgium	March 1	May 2	57	49,517	29,418	7765	0.99	May 4	0.86
Finland	February 26	April 29	57	4395	2500	177	0.83	April 15	1.03
Thailand	January 26	April 14	61	2613	1405	41	0.89	May 17	0.08
Israel	February 27	April 29	62	15,834	8233	215	0.95	May 4	0.58
France	February 6	April 26	69	162,220	98,853	22,859	0.91	May 11	0.50
Spain	February 25	May 6	71	220,325	126,002	25,857	0.98	June 20	0.85
Sweden	February 26	May 9	73	25,921	14,957	3220	0.98	—^b^	—
Hungary	March 14	June 10	88	198,811	69,957	23,633	0.98	May 28	1.21
Italy	February 22	May 20	89	228,006	134,560	32,486	0.93	May 18	1.07
Japan	January 26	May 11	90	450,249	94,167	13,001	0.99	May 25	0.28
Singapore	February 8	May 26	90	32,343	16,444	23	0.97	June 6	0.55
Russia	March 5	June 10	97	493,023	6350	252,295	0.98	June 9	1.03
Canada	February 12	June 2	112	94,641	51,506	7579	0.99	June 1	1.03
Philippines	—	—	—	—	—	—	—	May 15	4.27
United States	—	—	—	—	—	—	—	May 1	6.18

^a^LSD: lifting social distancing.

^b^Not available.

### Resurgence of the COVID-19 Epidemic in Local Communities and Regions

The LSD index can also be applied to evaluating whether social distancing has to be re-executed in local cities and regions because of COVID-19 outbreak resurgences due to small cluster infections. In the United States, states like Florida and Texas saw a resurgence of the COVID-19 epidemic after LSD. Using these two states as examples, although the overall LSD until the end of April was 0.83 (Table 2), the LSD indices from June 27 to July 4 were estimated as 0.94 and 1.15, respectively, indicating that the outbreak was re-emerging and might call for a restrengthening of social distancing measures. Similar resurgence can be noted in Texas.

**Table 2:** Resurgence of COVID-19 epidemic in Florida and Texas.

Date	Florida					Texas			
	COVID-19 cases	Recovered cases	Death cases	LSD^a^ index	COVID-19 cases		Recovered cases	Death cases	LSD index
April 11	18,494	438	3325	4.70	12,561		254	1617	6.93
April 18	25,269	754	10,357	1.51	18,260		453	4806	2.90
April 25	30,839	1075	17,419	0.83	23,773		623	9986	1.44
May 02	35,463	1388	23,881	0.55	30,522		847	14,891	1.11
May 09	40,001	1785	29,054	0.44	37,860		1049	20,141	0.93
May 16	44,811	2040	33,423	0.40	46,999		1305	26,601	0.82
May 23	50,127	2312	37,689	0.39	54,509		1506	32,277	0.74
May 30	55,424	2530	42,281	0.37	62,338		1626	40,068	0.60
June 06	62,758	2773	47,354	0.39	73,553		1819	48,895	0.54
June 13	73,552	3016	52,408	0.46	86,011		1957	56,535	0.56
June 20	93,797	3237	59,521	0.63	103,305		2140	65,329	0.61
June 27	132,545	3489	70,063	0.94	143,371		2366	78,248	0.86
July 04	190,052	3803	89,994	1.15	191,790		2608	97,430	1.00

^a^LSD: lifting social distancing.

### The LSD Index for Other Emerging Infectious Diseases

**Figure 5:**
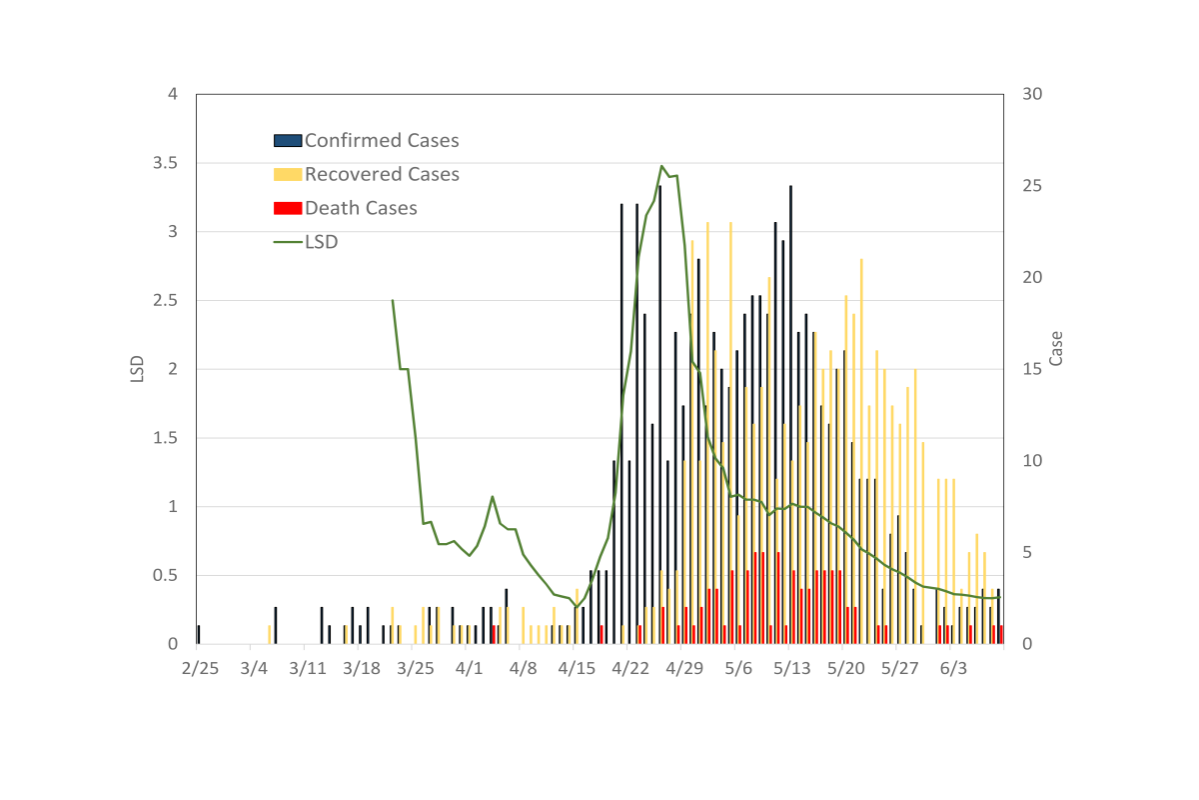
LSD index for severe acute respiratory syndrome outbreak in Taiwan, 2003. The 2.5 LSD index was high on March 22, 2003, at the initial epidemic stage then decreased to 0.3 on April 15, 2003. After April 22, the LSD index started increasing from 1 by nosocomial infection. The lower LSD index (<0.5) indicated that social distancing could have been lifted at the end of May. LSD: lifting social distancing.

The LSD index was also applied in a case of a SARS outbreak. During the 2003 outbreak of SARS in Taiwan, a total of 664 probable cases with 81 deaths were reported in the epidemic period between February 25 and June 15, 2003 (Figure 5). The majority of infected cases were imported as a minor outbreak in the initial period before April. The LSD index was as high as 2.5 on March 22 then down to 0.3 on April 15, 2003. Since April 22, 2003, the probable cases increased, and the LSD index rose to be greater than 1. The source of the outbreak was associated with health care settings. Several nosocomial SARS clusters were reported April 22-May 22, 2003. By a fever screen at emergency departments, self-quarantine, isolation, and containment of health care transmission, the spread of SARS was finally stopped. A low LSD index of 0.3 indicated that lifting of social distancing could have been considered at the end of May 2003.

**Figure 6:**
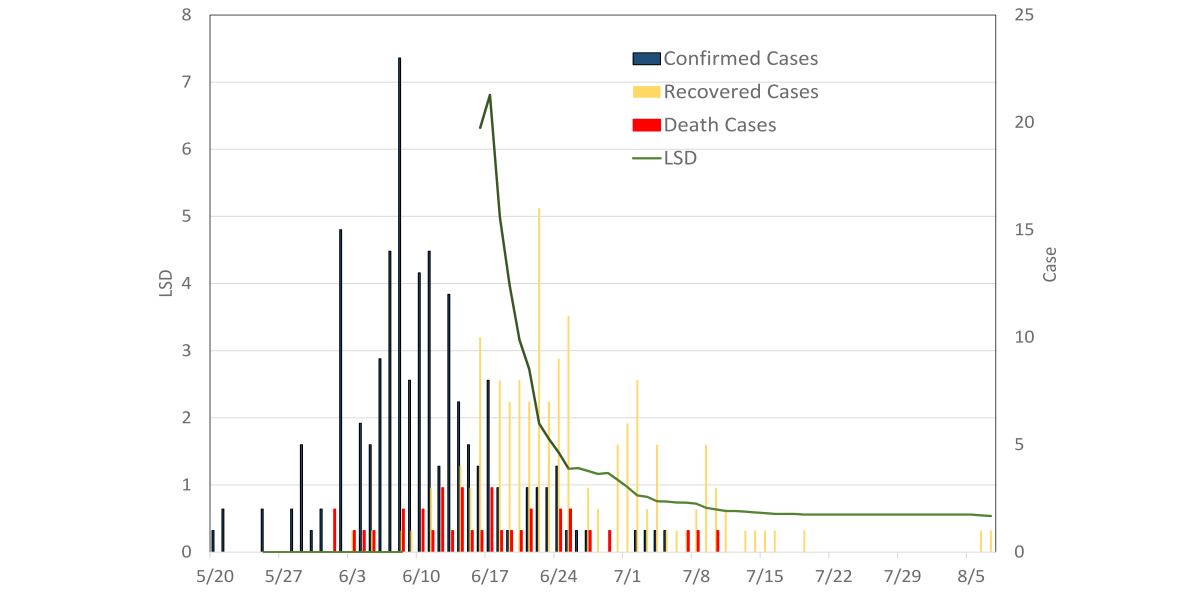
LSD index for the Middle East respiratory syndrome–related coronavirus outbreak in South Korea, 2015. The LSD index greater than 6 was estimated on June 16, 2015. After about 2 weeks, the LSD index was less than 1 on July 1, 2015, and kept at a low value until the end of the outbreak. LSD: lifted social distancing.

Figure 6 shows the estimated results of the LSD index applied to the 2015 MERS-CoV outbreak in South Korea. The outbreak started on May 20, 2015, with an imported case of MERS-CoV. Following the index case, the MERS-CoV outbreak in South Korea lasted until August 5, 2015, yielding 186 cases and 36 deaths. The LSD index for this outbreak was estimated since June 16, 2015, at the initial stage. The LSD index became less than 1 on July 1, 2015, around 2 weeks after the peak of 6.8 on June 17, 2015, and was kept at a low value until the end of the outbreak on August 7, 2015. This decreasing trend in LSD was attributable to the increasing recovery rate, which reached 80% after July 19, 2015, and the controlled case-fatality rate of 19% during the same period.

**Figure 7:**
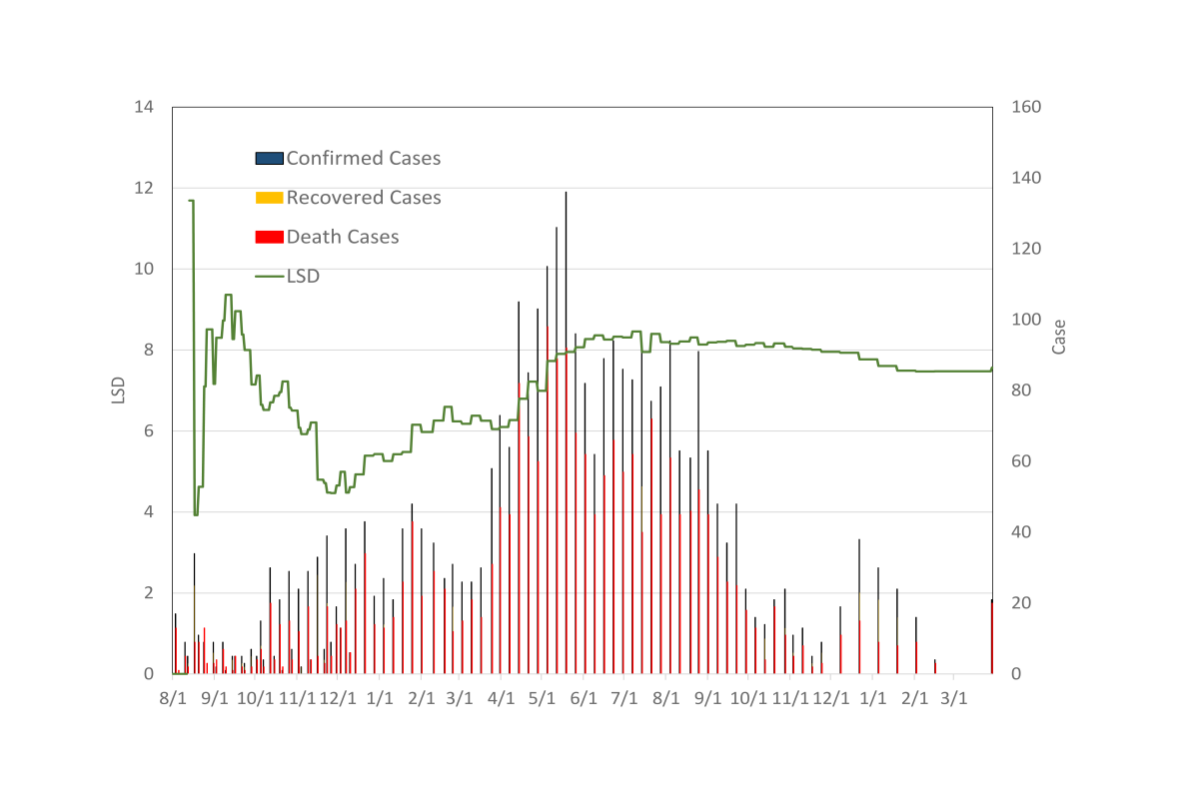
LSD index for the Ebola outbreak in the Democratic Republic of the Congo, 2018. The LSD index was estimated as 11.7 at the initial stage on August 12, 2018. The LSD index never became less than one. LSD: lifting social distancing.

Figure 7 shows the estimated results of the LSD index along with the frequencies of cases, deaths, and recoveries for the Ebola outbreak in the Democratic Republic of Congo since mid-2018. The end of the outbreak was declared on June 25, 2020, when the 42-day lapse between the last case was reached. The LSD index estimated on August 12, 2018, was 11.7 at the initial stage. As the Ebola epidemic evolved, the LSD index never became less than 1. This was not only due to its high case-fatality rate (55-79%) but also its lower recovery rate (<45%) through the whole epidemic. This high level of LSD revealed less effectiveness to depend on nonpharmaceutical approaches for containing a disease as lethal as Ebola.

## Discussion

The COVID-19 pandemic has evolved from the dawn of large-scale outbreaks followed by large-scale social distancing plans to contain the COVID-19 epidemic. Incorporating information on the effective social distancing interventions to reduce the contacts of adults and avert the hospitalizations and deaths into mathematical models for guidance on containment measures has been suggested [17,18]. However, to revive economic and social activity, LSD is still necessary and requires a simple index for guiding LSD particularly when a travel ban has been lifted across borders and regions. We propose a simple index for LSD for global, country, region, and community levels to elucidate the global dynamic change of three factors in relation to COVID-19 through three phases (the large-scale outbreak period [between January and early March], the pandemic period [between mid-March and June], and the postpandemic period [starting in June]) and the corresponding dynamic changes at the country and region level through three epidemic phases (pre-epidemic, epidemic, and postepidemic) of COVID-19 but with different time windows in each specific country and region. The LSD index has been applied to monitoring the resurgence of small to moderate-small cluster infections after LSD. There are three main merits of using this LSD index. First, the LSD index is informative in providing a quantitative assessment for whether and when to lift social distancing. Second, the LSD index identifies which factor of the three determinants may contribute more to a higher value of LSD than others, which provides information for health decision makers to reinforce this fragile determinant for shortening the time until the lifting of social distancing. Third, the LSD index can be flexibly applicable to various scenarios from local, country, and region to the global community during the postpandemic period and accommodate the regions that have already lifted social distancing but have seen resurgences of outbreaks due to cluster infections.

The dynamic change of weekly LSD indices on a global level is informative to get a better understanding of the time required for the change from LSD≥1 to LSD<1. The longer time required for LSD, the less likely the countries or regions will be resilient enough to recover from the COVID-19 pandemic. On the other hand, the shorter the time to reach an LSD less than 1, the more prosperous traveling business can be. The time required for countries or regions to change the value of LSD from greater than 1 to less than 1 ranged from 3 weeks to more than 4 months. Moreover, the LSD cutoffs for lifting can be rectified according to the fragile degree of public health in each country and region. The more fragile the public health system, the lower the suggested LSD cutoff.

Few countries took only 3 weeks for LSD, such as New Zealand. The countries that required 1-2 months for LSD were China, South Korea, and Vietnam in Asia; Iceland, Denmark, Austria, Ireland, Netherlands, Switzerland, Germany, and Turkey in Europe; and Australia in Oceania. By contrast, Thailand and Malaysia in South Asia; Israel in Western Asia; and Belgium, Finland, France, Sweden, and Spain in Europe required 2-3 months for LSD. Japan and Singapore in Asia and Italy, Hungary, and Russia in Europe required 3-4 months for LSD. Canada required 4 months. The time required for LSD is heterogeneous and is highly dependent on three determinants. In addition to maintaining containment measures, the reinforcement of the medical resource capacity is also essential to respond to the outbreak [20,23] when LSD.

For resource-limited countries, the LSD index did not decline much due to the insufficient medical resources. Following the WHO’s guidance [19] for patient triage and referral during community transmission will help health facilities cope with the COVID-19 pandemic to shorten the time for LSD. It is interesting to note that the LSD index for Ebola was always greater than 1, indicating that the outbreak was not able to lift social distancing until most of the infected died. This strongly suggested the necessity of discovering the vaccine for containing the epidemic of Ebola.

There is one limitation of using the LSD index. Complete and accurate information on confirmed cases, recoveries, and deaths is required for calculating the LSD index. There are some countries lacking the information on recoveries, which limits the use of the LSD index.

In summary, a simple index for LSD was developed to evaluate the evolution of this index through three phases, prepandemic, pandemic, and postpandemic, on the global level. The surveillance of LSD indices on the country and region level aids health policy makers in being aware of which epidemic phase they are in and assessing whether and when to lift social distancing in the postpandemic period. Decomposition of this index into three components also gives a clue to facilitating the process of LSD by improving the key determinant identified from the LSD index. The proposed LSD index for LSD can be applied to not only the current COVID-19 outbreaks but also other emerging infectious diseases in the future.

## Appendix

Multimedia Appendix 1 Supplementary material.

Multimedia Appendix 2 Dynamic change of global weekly lifting social distancing (LSD) index. Temporal change of global LSD index with 9 categories for 186 countries and regions from January 26 (fifth week) to July 5, 2020 (28th week). NA: not available.

## Abbreviations

CI: credible interval
CSSE: Center for Systems Science and Engineering
LSD: lifting social distancing
MERS-CoV: Middle East respiratory syndrome–related coronavirus
R_0_: basic reproductive number
SARS: severe acute respiratory syndrome
SEIRD: susceptible-exposed-infected-recovered-death
WHO: World Health Organization
