# Supplementary material for: An Index for Lifting Social Distancing During the COVID-19 Pandemic: Algorithm Recommendation for Lifting Social Distancing
Source: J Med Internet Res. 2020 Sep 17;22(9):e22469. doi: 10.2196/22469 (PMC7505695; doi:10.2196/22469)
Supplement: Multimedia Appendix 1 [file jmir_v22i9e22469_app1.docx]

Appendix

Appendix Figure 1. Compartment model with Susceptible (S), Exposed (E), Infected (I), Recovered (R), and Death (D), SEIRD model for the transmission and evolution of COVID-19. The notations of four parameters, β, α, γ, and τ represent contact rate, the progression rates to infective status, recovery rate, and case fatality rate, respectively.


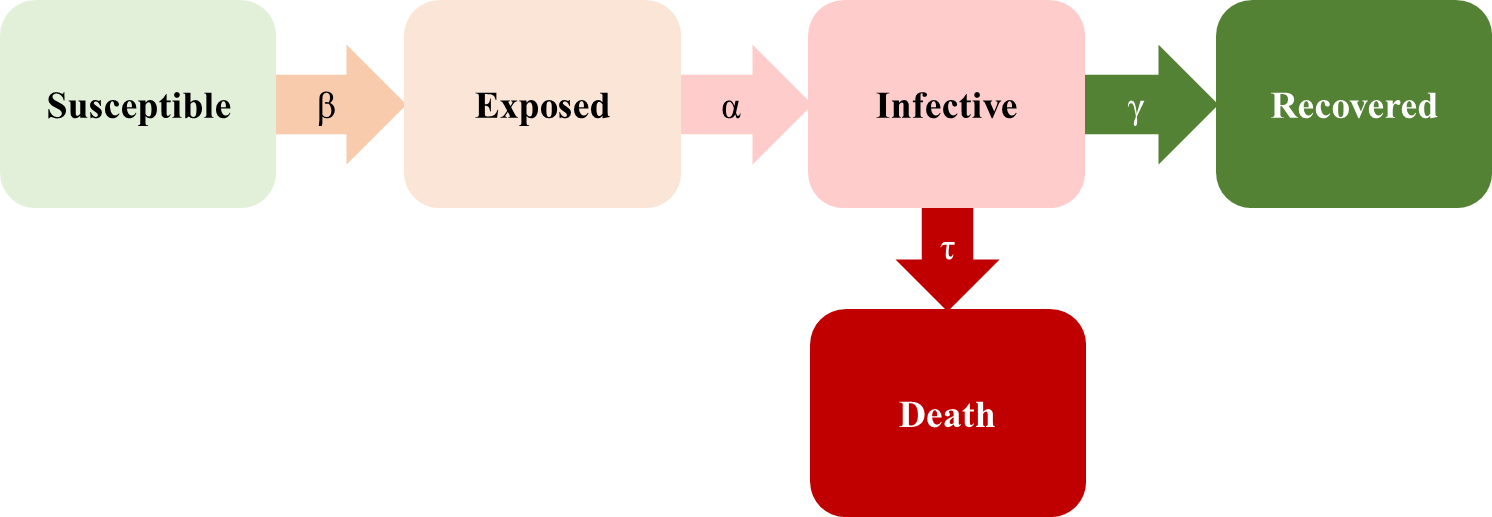


Appendix Table 1: Daily Covid-19 cases, recovered cases, death cases, and LSD

| **Date** | **Confirmed Cases** | **Recovered Cases** | **Death Cases** | **LSD** |
| --- | --- | --- | --- | --- |
| 22-Jan | 555 | 28 | 17 | 19.45 |
| 23-Jan | 654 | 30 | 18 | 21.42 |
| 24-Jan | 941 | 36 | 26 | 25.88 |
| 25-Jan | 1434 | 39 | 42 | 36.88 |
| 26-Jan | 2118 | 52 | 56 | 40.84 |
| 27-Jan | 2927 | 61 | 82 | 48.37 |
| 28-Jan | 5578 | 107 | 131 | 52.38 |
| 29-Jan | 6166 | 126 | 133 | 49.02 |
| 30-Jan | 8234 | 143 | 171 | 57.80 |
| 31-Jan | 9927 | 222 | 213 | 44.70 |
| 1-Feb | 12038 | 284 | 259 | 42.32 |
| 2-Feb | 16787 | 472 | 362 | 35.35 |
| 3-Feb | 19887 | 623 | 426 | 31.62 |
| 4-Feb | 23898 | 852 | 492 | 27.64 |
| 5-Feb | 27642 | 1124 | 564 | 24.10 |
| 6-Feb | 30801 | 1487 | 634 | 20.15 |
| 7-Feb | 34336 | 2013 | 719 | 16.42 |
| 8-Feb | 37069 | 2619 | 806 | 13.47 |
| 9-Feb | 40097 | 3247 | 906 | 11.63 |
| 10-Feb | 42633 | 3949 | 1013 | 10.06 |
| 11-Feb | 44674 | 4687 | 1113 | 8.78 |
| 12-Feb | 45053 | 5152 | 1118 | 7.97 |
| 13-Feb | 60201 | 6297 | 1371 | 8.78 |
| 14-Feb | 66676 | 8063 | 1523 | 7.46 |
| 15-Feb | 68754 | 9398 | 1666 | 6.50 |
| 16-Feb | 70878 | 10858 | 1770 | 5.69 |
| 17-Feb | 72814 | 12581 | 1868 | 4.94 |
| 18-Feb | 74604 | 14351 | 2007 | 4.34 |
| 19-Feb | 75029 | 16120 | 2122 | 3.79 |
| 20-Feb | 75576 | 18176 | 2245 | 3.29 |
| 21-Feb | 76199 | 18889 | 2249 | 3.16 |
| 22-Feb | 77952 | 22894 | 2456 | 2.52 |
| 23-Feb | 78286 | 23404 | 2466 | 2.45 |
| 24-Feb | 78851 | 25237 | 2626 | 2.23 |
| 25-Feb | 79702 | 27909 | 2705 | 1.96 |
| 26-Feb | 80665 | 30378 | 2766 | 1.75 |
| 27-Feb | 82028 | 33273 | 2810 | 1.55 |
| 28-Feb | 83401 | 36708 | 2866 | 1.35 |
| 29-Feb | 85300 | 39779 | 2935 | 1.22 |
| 1-Mar | 87678 | 42714 | 2990 | 1.13 |
| 2-Mar | 89651 | 45601 | 3079 | 1.04 |
| 3-Mar | 92227 | 48226 | 3154 | 0.98 |
| 4-Mar | 94529 | 51169 | 3248 | 0.91 |
| 5-Mar | 97317 | 53795 | 3341 | 0.87 |
| 6-Mar | 101265 | 55830 | 3453 | 0.88 |
| 7-Mar | 105298 | 58311 | 3552 | 0.87 |
| 8-Mar | 109257 | 60641 | 3795 | 0.87 |
| 9-Mar | 113138 | 62441 | 3981 | 0.88 |
| 10-Mar | 118174 | 64337 | 4257 | 0.91 |
| 11-Mar | 125840 | 66637 | 4604 | 0.96 |
| 12-Mar | 131534 | 67991 | 4906 | 1.01 |
| 13-Mar | 145966 | 69912 | 5404 | 1.17 |
| 14-Mar | 157067 | 72297 | 5823 | 1.26 |
| 15-Mar | 168248 | 75746 | 6462 | 1.31 |
| 16-Mar | 182887 | 77822 | 7141 | 1.45 |
| 17-Mar | 198713 | 80580 | 7947 | 1.57 |
| 18-Mar | 218277 | 83141 | 8842 | 1.74 |
| 19-Mar | 246193 | 84900 | 9947 | 2.02 |
| 20-Mar | 275763 | 87540 | 11428 | 2.29 |
| 21-Mar | 308078 | 91835 | 13133 | 2.50 |
| 22-Mar | 341540 | 96045 | 14833 | 2.72 |
| 23-Mar | 383692 | 96554 | 16750 | 3.16 |
| 24-Mar | 424823 | 105385 | 19018 | 3.22 |
| 25-Mar | 475657 | 110789 | 21793 | 3.50 |
| 26-Mar | 538595 | 117820 | 24800 | 3.79 |
| 27-Mar | 602962 | 127522 | 28314 | 3.96 |
| 28-Mar | 670595 | 136632 | 31993 | 4.15 |
| 29-Mar | 730166 | 144542 | 35461 | 4.31 |
| 30-Mar | 794711 | 161190 | 39623 | 4.19 |
| 31-Mar | 870960 | 174004 | 44467 | 4.27 |
| 1-Apr | 947119 | 188301 | 50014 | 4.31 |
| 2-Apr | 1028554 | 205040 | 56321 | 4.31 |
| 3-Apr | 1111494 | 220606 | 62301 | 4.34 |
| 4-Apr | 1192021 | 241191 | 68150 | 4.24 |
| 5-Apr | 1263388 | 256817 | 73165 | 4.22 |
| 6-Apr | 1336341 | 277158 | 79002 | 4.12 |
| 7-Apr | 1413091 | 301363 | 86909 | 4.00 |
| 8-Apr | 1496682 | 332879 | 93638 | 3.80 |
| 9-Apr | 1583123 | 362241 | 101253 | 3.67 |
| 10-Apr | 1671008 | 388253 | 108518 | 3.60 |
| 11-Apr | 1747502 | 421373 | 114573 | 3.44 |
| 12-Apr | 1844505 | 446293 | 120304 | 3.42 |
| 13-Apr | 1914421 | 482205 | 126057 | 3.25 |
| 14-Apr | 1984119 | 515282 | 132958 | 3.13 |
| 15-Apr | 2064845 | 548096 | 141257 | 3.04 |
| 16-Apr | 2161520 | 584123 | 148554 | 2.97 |
| 17-Apr | 2249845 | 619010 | 157442 | 2.91 |
| 18-Apr | 2323110 | 649598 | 163913 | 2.85 |
| 19-Apr | 2403952 | 679414 | 168491 | 2.81 |
| 20-Apr | 2476947 | 710699 | 173914 | 2.75 |
| 21-Apr | 2552062 | 751535 | 181065 | 2.66 |
| 22-Apr | 2628939 | 787526 | 187822 | 2.60 |
| 23-Apr | 2718376 | 823734 | 194691 | 2.55 |
| 24-Apr | 2805392 | 881144 | 201372 | 2.43 |
| 25-Apr | 2889583 | 914046 | 206946 | 2.41 |
| 26-Apr | 2962521 | 973112 | 210825 | 2.28 |
| 27-Apr | 3031782 | 1008012 | 215463 | 2.24 |
| 28-Apr | 3106364 | 1048256 | 221916 | 2.19 |
| 29-Apr | 3183241 | 1097988 | 228680 | 2.12 |
| 30-Apr | 3267019 | 1178848 | 234637 | 1.99 |
| 1-May | 3354010 | 1222512 | 239809 | 1.95 |
| 2-May | 3434560 | 1266900 | 245127 | 1.92 |
| 3-May | 3512302 | 1307058 | 248569 | 1.89 |
| 4-May | 3588412 | 1347267 | 252724 | 1.87 |
| 5-May | 3668451 | 1388943 | 258603 | 1.84 |
| 6-May | 3758372 | 1435314 | 265257 | 1.82 |
| 7-May | 3847584 | 1478721 | 270674 | 1.80 |
| 8-May | 3939112 | 1519217 | 276237 | 1.79 |
| 9-May | 4022720 | 1576584 | 280500 | 1.74 |
| 10-May | 4099011 | 1611908 | 284070 | 1.73 |
| 11-May | 4175285 | 1665243 | 287532 | 1.69 |
| 12-May | 4258682 | 1704199 | 293075 | 1.68 |
| 13-May | 4343700 | 1757768 | 298306 | 1.65 |
| 14-May | 4440778 | 1803203 | 303577 | 1.64 |
| 15-May | 4537158 | 1850673 | 308790 | 1.63 |
| 16-May | 4628951 | 1907373 | 312943 | 1.60 |
| 17-May | 4707484 | 1947161 | 316282 | 1.59 |
| 18-May | 4795755 | 1997772 | 319569 | 1.57 |
| 19-May | 4892213 | 2047693 | 324360 | 1.56 |
| 20-May | 4995021 | 2103713 | 329221 | 1.54 |
| 21-May | 5101557 | 2152305 | 334013 | 1.54 |
| 22-May | 5208523 | 2257306 | 339299 | 1.47 |
| 23-May | 5307504 | 2311939 | 343290 | 1.45 |
| 24-May | 5402803 | 2364666 | 346112 | 1.44 |
| 25-May | 5489776 | 2430477 | 347294 | 1.41 |
| 26-May | 5582310 | 2488382 | 351505 | 1.39 |
| 27-May | 5684912 | 2550175 | 356692 | 1.38 |
| 28-May | 5802878 | 2614348 | 361377 | 1.37 |
| 29-May | 5923988 | 2677191 | 366061 | 1.36 |
| 30-May | 6052413 | 2740440 | 370196 | 1.35 |
| 31-May | 6159522 | 2815632 | 373063 | 1.33 |
| 1-Jun | 6255772 | 2874676 | 376593 | 1.32 |
| 2-Jun | 6368325 | 2974045 | 381292 | 1.28 |
| 3-Jun | 6495660 | 3051246 | 386998 | 1.26 |
| 4-Jun | 6622362 | 3114493 | 392146 | 1.26 |
| 5-Jun | 6755003 | 3182550 | 396968 | 1.26 |
| 6-Jun | 6880809 | 3252291 | 400786 | 1.25 |
| 7-Jun | 6993249 | 3307740 | 403535 | 1.24 |
| 8-Jun | 7096688 | 3372076 | 407291 | 1.23 |
| 9-Jun | 7219671 | 3442433 | 412146 | 1.22 |
| 10-Jun | 7353451 | 3520692 | 417351 | 1.21 |
| 11-Jun | 7491639 | 3599585 | 422135 | 1.21 |
| 12-Jun | 7620625 | 3679723 | 426430 | 1.19 |
| 13-Jun | 7754724 | 3763807 | 430680 | 1.18 |
| 14-Jun | 7887854 | 3831475 | 434026 | 1.18 |
| 15-Jun | 8009639 | 3914359 | 437530 | 1.16 |
| 16-Jun | 8149218 | 3997000 | 444319 | 1.16 |
| 17-Jun | 8325110 | 4103986 | 449590 | 1.14 |
| 18-Jun | 8463985 | 4187036 | 454599 | 1.14 |
| 19-Jun | 8644919 | 4283155 | 460868 | 1.13 |
| 20-Jun | 8803183 | 4384841 | 465136 | 1.12 |
| 21-Jun | 8934086 | 4457206 | 469193 | 1.12 |
| 22-Jun | 9071945 | 4543423 | 472764 | 1.11 |
| 23-Jun | 9237292 | 4633281 | 478188 | 1.10 |
| 24-Jun | 9404928 | 4731705 | 483362 | 1.10 |
| 25-Jun | 9582887 | 4826517 | 489913 | 1.09 |
| 26-Jun | 9773888 | 4937457 | 494758 | 1.09 |
| 27-Jun | 9951702 | 5048068 | 499276 | 1.08 |
| 28-Jun | 10116920 | 5134265 | 502435 | 1.07 |
| 29-Jun | 10272789 | 5224574 | 506061 | 1.07 |
| 30-Jun | 10447158 | 5337367 | 511253 | 1.06 |
| 1-Jul | 10663447 | 5451393 | 516238 | 1.06 |
| 2-Jul | 10871205 | 5616200 | 521346 | 1.03 |
| 3-Jul | 11075365 | 5728149 | 526404 | 1.03 |
| 4-Jul | 11268393 | 5940536 | 530752 | 0.99 |
| 5-Jul | 11451475 | 6037076 | 534265 | 0.99 |
