# Supplementary figures and images for: An Index for Lifting Social Distancing During the COVID-19 Pandemic: Algorithm Recommendation for Lifting Social Distancing
Source: J Med Internet Res. 2020 Sep 17;22(9):e22469. doi: 10.2196/22469 (PMC7505695; doi:10.2196/22469)

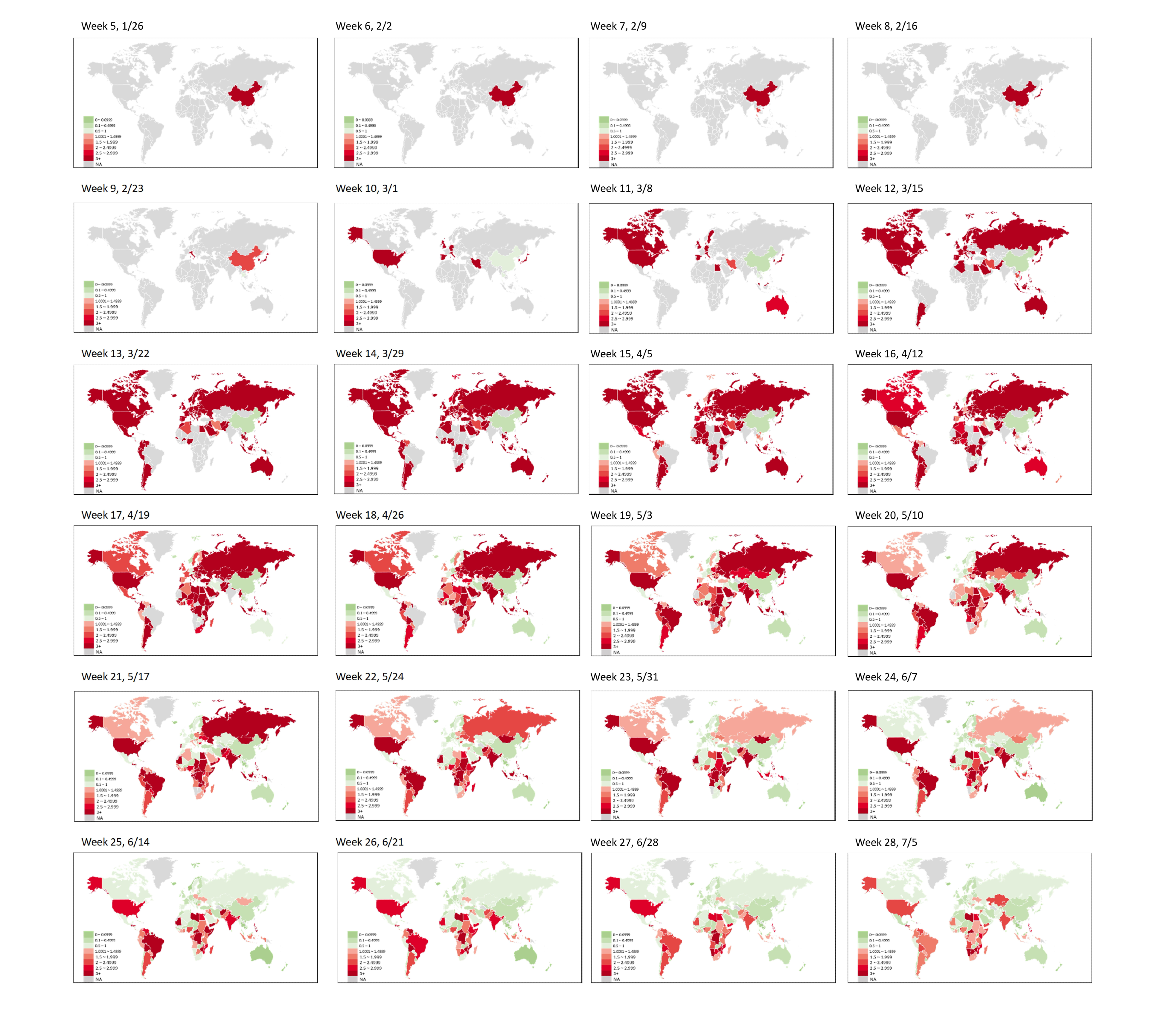

Supplement: Multimedia Appendix 2 [file jmir_v22i9e22469_app2.png]
